# The effects of parental components in a trauma-focused cognitive behavioral based therapy for children exposed to interparental violence: study protocol for a randomized controlled trial

**DOI:** 10.1186/s12888-015-0533-7

**Published:** 2015-06-23

**Authors:** Margreet M. Visser, Machteld D. Telman, J. Clasien de Schipper, Francien Lamers-Winkelman, Carlo Schuengel, Catrin Finkenauer

**Affiliations:** 1 KJTC (Children’s Trauma Center Haarlem), Zuiderhoutlaan 12, Haarlem, 2012 PJ The Netherlands; 2 Department of Clinical Child and Family Studies, VU University Amsterdam, Van der Boechorststraat 1, 1081 BT Amsterdam, The Netherlands; 3 EMGO Institute for Health and Care Research, Van der Boechorststraat 7, Amsterdam, 1081 BT The Netherlands

**Keywords:** Children, Posttraumatic stress disorder, Trauma-focused cognitive-behavioral-therapy, Interparental violence, Domestic violence, Randomized clinical trial, Parental availability, Parent-child interaction

## Abstract

**Background:**

Interparental violence is both common and harmful and impacts children’s lives directly and indirectly. *Direct effects* refer to affective, behavioral, and cognitive responses to interparental violence and psychosocial adjustment. *Indirect effects* refer to deteriorated parental availability and parent-child interaction. Standard Trauma Focused Cognitive Behavioral Therapy may be insufficient for children traumatized by exposure to interparental violence, given the pervasive impact of interparental violence on the family system. HORIZON is a trauma focused cognitive behavioral therapy based group program with the added component of a preparatory parenting program aimed at improving parental availability; and the added component of parent-child sessions to improve parent-child interaction.

**Methods/design:**

This is a multicenter, multi-informant and multi-method randomized clinical trial study with a 2 by 2 factorial experimental design. Participants (*N* = 100) are children (4–12 years), and their parents, who have been exposed to interparental violence. The main aim of the study is to test the effects of two parental components as an addition to a trauma focused cognitive behavioral based group therapy for reducing children’s symptoms. Primary outcome measures are posttraumatic stress symptoms, and internalizing and externalizing problems in children. The secondary aim of the study is to test the effect of the two added components on adjustment problems in children and to test whether enhanced effects can be explained by changes in children’s responses towards experienced violence, in parental availability, and in quality of parent-child interaction. To address this secondary aim, the main parameters are observational and questionnaire measures of parental availability, parent-child relationship variables, children’s adjustment problems and children’s responses to interparental violence. Data are collected three times: before and after the program and six months later. Both intention-to-treat and completer analyses will be done.

**Discussion:**

The current study will enhance our understanding of the efficacy interparental violence-related parental components added to trauma focused cognitive behavioral group program for children who have been exposed to IPV. It will illuminate mechanisms underlying change by considering multiple dimensions of child responses, parenting variables and identify selection criteria for participation in treatment.

**Trial registration:**

Netherlands Trial Register NTR4015. Registered 4th of June, 2013.

## Background

Interparental violence (IPV) is both common and harmful. At least 12 % of 12–16 year old children are exposed to IPV in The Netherlands [1]. In the United States, 16 % of all children witness IPV at some time during their childhood (2–17 years of age) [2]. In a meta-analysis Evans [3] found a strong association between exposure to IPV and trauma symptoms in children, in addition to small to medium associations between exposure to IPV and internalizing and externalizing problems. These findings emphasize the need for effective interventions for children exposed to IPV. Because IPV involves the whole family system, it affects children’s lives directly and indirectly. Witnessing IPV or being physically involved in IPV may directly affect children’s affective, behavioral and cognitive responses, their psychosocial adjustment and symptoms [4]. IPV may also affect children indirectly [4]. It may lead to deteriorated parenting and parent-child relationships [5], which may mediate the link between IPV and children’s maladjustment on various dimensions. Therefore, treatment for children who have been exposed to IPV should target both direct and indirect effects of IPV.

Trauma Focused-Cognitive Behavioral Therapy (TF-CBT) is a well-established treatment for traumatized children. Although TF-CBT has been found to be effective in reducing post-traumatic stress disorder and depressive symptoms among traumatized children [6], we know little about its effective components and the role of parental involvement [7]. Additionally, the literature suggests that TF-CBT may be less effective for children traumatized by exposure to IPV [8, 9]. One explanation may be that the standard components of TF-CBT have been developed for parents to learn how they can help the child to process traumatic experiences. These components may fail to address the pervasive impact of IPV on parents’ psychological functioning, their parental behavior, and the parent-child relationship [8]. The present study aims to test the relative efficacy of two components added to TF-CBT focussing on parent-related aspects of IPV, namely parenting and parent-child interactions, and thereby provides crucial insight in the mechanisms and mediating effects of treatment on children exposed to IPV. The group-based treatment developed by Visser, Leeuwenburgh and Lamers-Winkelman in the Netherlands is called HORIZON [10]. In addition to a regular TF-CBT-based treatment, the HORIZON includes two specific components focusing on parents who have let their children to become exposed to IPV. The HORIZON thus consists of three parts, two specific parental components for IPV families and TF-CBT-based child and parent components.

### Direct effects of IPV on children

Being exposed to IPV affects children on a variety of dimensions. Children’s responses are often differentiated in *emotional*, *behavioral*, and *cognitive* responses [11]. To explain these direct effects, Emotional Security Theory [4] and Cognitive Context theory [12] have proposed several mechanisms that mediate the developmental pathways towards psychosocial maladjustment and symptoms, as well as moderating factors that may exacerbate or buffer against the effects of IPV on children.

*Emotional Security Theory* is based on the assumption that children derive a sense of emotional security from their trust in the integrity of the family system. IPV undermines children’s trust, leading them to make efforts to restore it. Although children’s affective, behavioral, and cognitive responses and adaptations toward restoring a level of emotional security may be adaptive in the IPV context, they may be maladaptive in other contexts (e.g., school, peer contacts). This may result in emotional, social, and behavioral maladjustment and problems [13]. To illustrate, Davies et al. [14] and Katz et al. [13] found that children exposed to IPV were less competent in modulating their emotions than children who were not exposed to IPV. This under-developed emotion competence may explain links between IPV and children’s maladjustment [13].

Children’s immediate *behavioral* responses to IPV include approach and avoidance behaviors that serve to regulate exposure to the disturbing affect displayed in marital violence and its aftermath [15, 16]. Avoidance behavior is generally considered as a less adaptive coping strategy to respond to traumatic experiences than approach behavior [17]. To illustrate, Gable [18] found that approach motives and goals in relationships were reliably associated with less loneliness and more relationship satisfaction than avoidance motives. Although not consistently found, both approach and avoidance behaviors in the context of family conflict appear related to children’s symptoms [11].

Children’s *cognitive* responses to violence may shape their beliefs and expectations about aggression, about close relationships and about themselves. They might start to believe that aggressive behavior is an acceptable way of problem solving [19]. Children exposed to IPV and other forms of violence may come to value aggression more positively than other children [20, 21]. Additionally, Grych’s Cognitive Context theory suggests that children may blame themselves for what happened or believe they are powerless to cope with IPV [22]. Finally, children may become more vigilant to threat-related cues in the environment [23]. These beliefs and expectations may influence their behavior in peer-relationships, in family-relationships and in romantic relationships [22]. Each of these cognitive responses of children can be assumed to exacerbate children’s psychosocial maladjustment in response to IPV.

Components of TF-CBT such as emotion regulation, cognitive reprocessing, psycho-education and skill building have been shown to reduce internalizing problems, externalizing problems, and trauma symptoms [9, 24]. Nevertheless, to our knowledge research has not yet explicitly addressed whether TF-CBT or TF-CBT-based interventions ameliorates children’s symptoms by improving children’s emotion regulation. Furthermore, TF-CBT was found to reduce trauma-related avoidance behavior in children exposed to IPV [9]. Whether children’s approach behavior changes after TF-CBT or TF-CBT-based interventions is currently unknown. Also, the cognitive component of TF-CBT explores and corrects children’s harmful attributions about the cause of, responsibility for, and results of traumatic experiences such as family violence [17, 25]. Whether cognitive responses change after TF-CBT among children who have been exposed to IPV has not yet been examined. The present study examines how these emotional, behavioral, and cognitive responses change over the course of treatment and how these changes affect treatment outcomes and children’s psychosocial adjustment.

### Indirect effects of IPV on children

Being exposed to IPV also affects children indirectly. Not only children, but parents are likely to be traumatized as well [26]. Parents experience a broad range of emotional, cognitive and behavioral consequences of IPV. These responses can be assumed to affect their parenting and the parent-child relationship. The *Spillover hypothesis* emphasizes that distressing experiences in the interparental relationship, such as IPV, carry over to parenting behavior [27] and to the parent-child interaction (e.g., [28]). We propose two mechanisms that may explain how the experience of IPV may affect parenting, a cognitive-emotional mechanism and a behavioral mechanism. In the following, we will describe these theoretical underpinnings of the two specific parental components of the HORIZON.

### Cognitive-emotional mechanism: parental availability

Mothers who have been part of IPV tend to underestimate the extent to which their child has been exposed to and is affected by the IPV [29, 30]. This underestimation is assumed to be partly due to the fact that mothers may focus their attention on themselves and their own traumatic experience rather than on their children’s traumatic experience [29, 30], and to the fact that their children’s behavior is a reminder of their own trauma which triggers avoidance e.g. [31]. Also, mothers who have experienced traumas, such as IPV or childhood abuse, showed difficulties in adopting an open, non-defensive style of communication when talking about emotions with their children [32]. Additionally, they appeared less child-centered and less available to their children [33]. Moreover, IPV has been linked to parenting styles that are characterized by emotional unavailability and psychological control e.g., [34, 35]. Thus, parents from violent families may be so absorbed by their own problems that they are less likely to take their children’s perspective and to show insight in their children’s developmental needs, behavior, and motives [36]. This deteriorated insightfulness may partly explain why children traumatized by exposure to IPV benefit less from TF-CBT than children who were traumatized by experiences that did not have a traumatic impact on their parents at the same time [8, 9].

For children to feel secure and supported by their parents, parents need to be sensitive to their children’s needs, accurately recognize their children’s signals, and appropriately respond to these signals [37, 38]. Similarly, for children to benefit from a trauma-focused treatment, such as TF-CBT, parental involvement to support the child is necessary [39]. Accordingly, increasing parents’ cognitive and emotional availability to the child should be a primary focus of treatment for children exposed to IPV. To this end, Visser, Leeuwenburgh, and Lamers-Winkelman developed a preparatory program for parents exposed to IPV [10]. This preparatory program was aimed to increase parental availability and insightfulness in their children’s needs. Parents are coached to enhance reading their children’s behavioral and emotional signals and to adequately interpret these signals in light of the child’s age, the development, and type of IPV the child has been exposed to. The preparatory program component precedes the TF-CBT-based treatment.

### Behavioral mechanism: parent-child interaction

Parents who have experienced and have been exposed to IPV tend to engage in ineffective parenting. Compared to non-exposed parents they used more negative and less positive parenting, are likely to use more harsh discipline towards their children [40], showed more aggression in the parent-child relationship [41], and were less supportive and less effective [42]. Buehler and Gerard [5] found that ineffective parenting mediated the link between marital conflict and children’s maladjustment. In their study, ineffective parenting comprised of harsh discipline (e.g., spanking), low involvement (e.g., talking and reading with child), and reduced parental presence (i.e., time spent together). These findings suggest that IPV reduces parents’ capacity to support their child, to interact with him/her in a safe and comforting manner, and to be available to fulfill and sensitively respond to his emotional and cognitive needs [43].

To optimally benefit from trauma-focused treatment, parental support is important for children [39]. Accordingly, we propose that trauma-focused treatment for children exposed to IPV can be enhanced by improving parents’ emotional support and parent-child interaction. To this end, Visser et al. [10] developed the second specific parental component, the parent-child interaction sessions, to complement the TF-CBT-based treatment. These weekly sessions follow the child and parent TF-CBT-based treatment sessions. They aim to help parents to gain new insights in their children’s functioning and to more accurately and positively respond to their child’s emotions and behaviors by interacting with their child.

### Trial objectives

In the present project, we will examine the efficacy of the two parental components of the HORIZON, the preparatory program for parents and the parent-child interaction sessions. HORIZON is a TF-CBT-based group treatment for children exposed to IPV. For a complete description of the treatment in Dutch see [10, 44], and for a summary see paragraph ‘Intervention’. In a randomized-controlled design, we will add the parental components to a TF-CBT-based core treatment to examine their independent and combined effects on parents’ and children’s outcomes. Overall, we expect both parental components to add to the efficacy of the TF-CBT-based core treatment. Specifically, we expect that, as compared to parents who did not participate in the preparatory program, parents who participated in the preparatory program show a greater increase in parental availability. In the same vein, we expect that the parents who participated in the parent-child interaction sessions show a greater increase in effective and positive parenting and a greater decrease in negative parenting than parents who did not participate in these sessions. These effects of the parental components should contribute to parents’ and children’s outcomes and reduction of symptoms.

### Primary objectives

The primary objective of the present study is to examine the efficacy of two parental components that complement a TF-CBT-based core treatment for children who have been exposed to IPV. Specifically, the study involves a randomized controlled trial with a 2 (parent preparatory program present vs. absent) X 2 (parent-child interaction sessions present vs. absent) factorial design to evaluate the effects of these two parental treatment components on child symptoms.

### Secondary objectives

Our second goal is to evaluate the effects of these two parental treatment components on child adjustment. Our third goal is to investigate mechanisms underlying the efficacy of treatment for children who have been exposed to IPV by examining associations between: child symptoms, on the one hand, and, 1) child responses (i.e., child emotional, behavioral, and cognitive responses, 2) parental availability and 3) parent-child interaction, on the other.

Our fourth goal is to examine specific hypotheses of change. Specifically, we will test whether, as predicted, the preparatory program leads to increased parental availability and whether the parent-child interaction sessions lead to improved parenting behavior. Also, we will explore whether these changes lead to a reduction in symptoms.

To ensure comparability of the randomized conditions, we will control for duration and severity of the IPV [45], parental psychopathology [46], and new incidents of IPV.

## Methods

### Study design

**Fig. 1:**
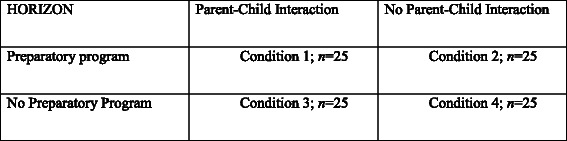
Study Design. Random-controlled trial examining the effectiveness of two parental components

**Fig. 2:**
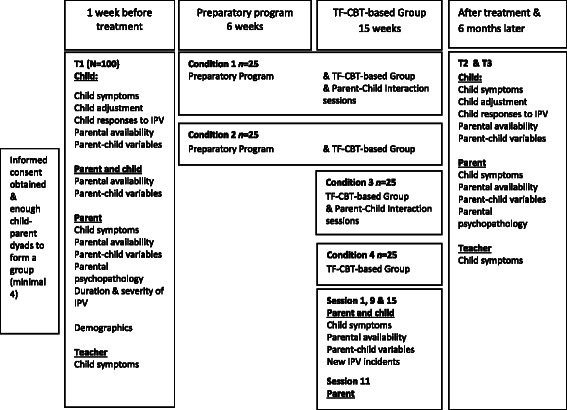
Research procedure. After informed consent is obtained for all parent-child dyads to form a group, they will all participate in the T1 assessments. Parents and children are asked to fill out questionnaires and to participate in two observational tasks. Additionally, the parent is interviewed with respect to the observational tasks. After T1, the group will be randomly assigned to one of the four treatment conditions by an independent researcher using a randomization procedure with lottery drawings. Condition 1 & 2 will start one week after T1, and condition 3 & 4 will start seven weeks after T1. One week (T2) and six months (T3) after the end of the program, parents and children are again invited to fill out questionnaires. At all assessments, the teacher is also sent a questionnaire

This multi-center study examines the addition of two parental components to a TF-CBT-based treatment for children exposed to IPV, which results in a 2 (preparatory program present versus absent) x 2 (parent-child interaction present versus absent) factorial randomized experimental component trial. The study includes pre-treatment (T1), treatment, post-treatment (T2), and a 6-month follow-up (T3) assessment, and will include 100 children and their custodial parents (Fig. 1 depicts the study design). The baseline assessment (T1, see Fig. 2) will take place one week prior to the start of the 6-week preparatory program. To ensure comparability across treatment conditions, in the “No preparatory program” condition, parents and children will be assessed 7 weeks before the beginning of the TF-CBT-based treatment. Additionally, parents and children will be assessed three times during treatment, namely at the beginning of the intervention (session 1), after sharing the trauma narrative of the child with the parent (session 9), and at the end of the intervention (session 15). These measurements allow us to test mediating pathways [47], and allow us to monitor, and if necessary control for, new IPV or other stressful incidents. The second assessment (T2) will take place one week after the last session of the TF-CBT-based treatment for all four conditions. The third assessment (T3) will be at a follow-up, six months after the last session of the treatment. Regardless of condition, all participants receive the standard TF-CBT-based treatment. The difference lies in the addition of the two parental components. Families who are assigned to “No preparatory program“ or “No parent-child interaction” conditions will not receive an alternative component additionally to standard TF-CBT based treatment.

### Randomization, blinding, and treatment allocation

Recruitment will take place in three mental health centers in The Netherlands. For each center families are referred for treatment after children have been exposed to interparental violence. As soon as a group of approximately 8 families have met the inclusion criteria for participation in the HORIZON group treatment (see Inclusion and Exclusion Criteria), and after informed consent is obtained, the families participating in the study will participate in T1 measurements. Parents and children are asked to fill out questionnaires and to participate in two observational tasks. Additionally, the parent is interviewed about the observational tasks. After T1, the group will be randomly assigned to one of the four treatment conditions (see Fig. 1) by an independent researcher using a randomization procedure with lottery drawings. Conditions 1 & 2 will start one week after T1, and conditions 3 & 4 will start seven weeks after T1. One week (T2) and six months (T3) after the end of the program, parents and children are again invited to fill out questionnaires (see Fig. 2). At all assessments the teacher is also sent a questionnaire. Typically, the intervention group consists of two child groups (4–7 years and 8–12 years, respectively) and one parent group. Usually there is only one parent group, because families often have more than one child who participate in the intervention, and because parents without visitation rights do not participate. The child groups receive treatment at the same time but in different rooms. The parents of children in both groups receive group sessions on the same time. Because it is not feasible for participating trauma centers to start four interventions at the same time, we cannot randomly assign individual children or parents to one of the four conditions. Therefore, we choose randomization by group just before the start of the group therapy and after T1. Parents and children are blind to treatment condition until randomization is carried out just before the (preparatory) program starts. Clinicians will be blind for treatment condition until all families are indicated for treatment. The assessments include videotaped observational tasks. Independent research assistants will code these videos. All coders are blind for treatment condition. Sometimes it will take a few months before families can participate in one of the four group interventions, due to waiting lists and enrollment numbers to form treatment groups. If necessary, parents and children will receive family or individual stabilization intervention during this time. Independent of their experiences with treatment preceding participation in the HORIZON, all participating families will be measured at T1 at the same time to ensure comparability of the families in the study.

### Study population

The population of this study will consist of 100 children exposed to IPV and their custodial parent who are referred by the Dutch Youth Care Agency (Bureau Jeugdzorg) or a physician for treatment of the child after exposure to IPV.

### Inclusion and exclusion criteria

Typically, children are referred to the HORIZON treatment when they meet the following criteria established during an intake-interview with a trained therapist and standardized questionnaires: 1) the child has been exposed to IPV (or violence between a parent and a cohabitant); 2) the child is no longer exposed to IPV (or violence between a parent and a cohabitant) 3) the child is between 4 and 12 years old; 4) both custodial parents gave written informed consent consistent with the Dutch legislation; 5) the child has trauma symptoms or behavioral problems; 6) the child can control his or her (sexual) impulses; 7) the child’s behavior is not dangerous to other children; 8) both child and custodial parent have sufficient cognitive and language capacities to follow a group treatment; 9) at least one custodial parent is able to participate in the parent-group.

If parents are unable to participate in the group therapy, for example, because they do not speak sufficient Dutch and have to receive individual treatment potentially with the help of a translator, parent and child will be excluded from the study. Children with severe psychopathology who represent a danger to other children receive individual treatment to stabilize their psychopathological problems. When stabilization is completed, children can participate in group therapy and will be included in the study.

### Procedure

The inclusion of children and parents in this study is bound to legal requirements to obtain informed consent from both parents before children can be enrolled in a research study. This requirement is problematic when permission needs to be asked for treatment and research independently (e.g., mothers may refuse contact with father). Therefore, we ask both parents’ permission for treatment and participation in the study for the child at the same time. Another challenge is that in the context of domestic violence, parents typically argue about almost everything, including consent for treatment and research. Consequently, when interpreting the findings and response percentages, the requirements to obtain consent from both parents should be taken into account.

### Objection by minors or incapacitated subjects

The code of conduct for minors in non-therapeutic research is applicable in this research project. The risk for participating in this project is considered negligible, but when a child seems adversely affected by the questionnaires or observational tasks it may be decided to (temporarily) discontinue participation in the project.

### Intervention

The HORIZON [10, 44] is a 21-session TF-CBT-based group intervention for children who have been exposed to IPV. The aim of the intervention is to help children process the traumatic experiences of having been exposed to IPV. The aim of the parent group is to guide parents to helping their traumatized children in this process. Both children and parents receive a therapy book [48, 49]. This book is used weekly during the therapy sessions for information about the topic, assignments, and drawings and in between sessions for homework.

For the description of the intervention we distinguish three parts. First, the *Preparatory Program* is developed for parents and consists of six sessions. As mentioned above, it aims to increase parental availability and insightfulness in children’s needs. Parents are coached to accurately read the behavioral and emotional signals of their children’s needs, and to adequately respond to these signals.

The second part of the intervention consists the TF-CBT based core program, which comprises of parallel groups for parents and children and consists of fifteen weekly sessions. Because the HORIZON is trauma-focused and based on cognitive behavioral therapy principles, it includes similar components as TF-CBT that were described and studied by Cohen and Mannarino [17]. Specifically, it includes components such as psycho-education, relaxation, affective expression and modulation, cognitive coping and processing, trauma narrative, sharing the trauma narrative with their (non-violent) parent and parenting skills. Similar to TF-CBT these components are covered in the HORIZON by the following exercises and themes: psycho-education about therapy, violence and conflicts, and posttraumatic stress; training of emotion regulation skills; addressing incorrect attributions about conflict and violence; expressing and sharing the IPV experiences; managing anger, guilt and shame, handling nightmares; good and bad sides of mother and father; and future safety. These weekly sessions have a duration of 60 min. After each session, the therapists of both the parent and child groups will evaluate the session and share information about children’s as well as parents’ progress.

The third part, the Parent Child Interaction Sessions (PCIS), takes place adjacent to the parallel parent and child group sessions when the parent group joins the children’s group. During 30-min sessions, parents and children are given the opportunity to interact with each other. The aim of these sessions for the parents is to learn to show more emotional supportive behavior, more involvement (e.g., talking together), more praise, less harsh discipline, and increase parental presence (e.g., time spent together). During Parent Child Interaction Sessions, parents can train and practice this parenting behavior through exercises in the presence of a therapist. Additionally, therapists observe the parent-child interaction and intervene when necessary. Therapists also give parents feedback on their parenting behavior in Parent Child Interaction Sessions during the following session in the parallel parent group. Children will receive feedback on their interaction behavior directly during the sessions together with their parents.

During the study, all children and parents in all four conditions will receive the TF-CBT based core treatment as described in part two above. There is no waiting list or control intervention.

## Measures

### Primary outcome measures

#### Child symptoms

##### Trauma symptoms

*Trauma Symptom Checklist for Younger Children* [50]. The TSCYC is a parent-reported questionnaire for children (3–12 years) measuring posttraumatic stress symptoms on a 4-point Likert scale, ranging from ‘not at all’ (1) to ‘very often’ (4). It consists of 90 items and 11 scales: two scales to assess the validity of the parent’s answers (response level and atypical response), eight clinical scales (anxiety, depression, aggression, PTSS-intrusion, PTSS-avoidance, PTSS-arousal, dissociation and sexual concerns) and a total PTSS score. This total score will be used in analyses. This clinical total PTS scale showed good reliability within a sample of maltreated children in the United States (Cronbach’s α = 0.81–0.91) and in the Netherlands (Cronbach’s α = 0.79–0.91) [51].

*Trauma Symptom Checklist for Children* [52]; Dutch translation: *Trauma Symptoom Controle Lijst voor Kinderen* [53]. This is a questionnaire to assess self-reported posttraumatic stress symptoms at children (8–12 years). It consists of 54 items clustering in 8 scales: two validity scales (underresponse, hyperresponse) and six clinical scales (anxiety, depression, post-traumatic stress disorder, dissociation, anger and sexual concerns). The response categories are the same as in the Trauma Symptom Checklist for Younger Children and reliability was high for the clinical total PTS score, with a Cronbach alpha’s ranging from 0.78 to 0.86 in a sample of sexually abused children [50]. In a sample of maltreated children in the United States the Trauma Symptom Checklist for Children showed discriminant and convergent validity with the Trauma Symptom Checklist for Younger Children [54], and in the Netherlands the Trauma Symptom Checklist for Children showed convergent and criterion validity with other behavioral questionnaires (CBCL, TRF, YSR, CDI) [55].

#### Internalizing and externalizing symptoms

*Child Behavior Checklist* [56] measures competencies and problem behaviors of children aged 1½ to 18 years. The CBCL has a parent-report and a teacher-report (TRF) questionnaire for 1½–5 years and 6–18 years. The questionnaire measures internalizing (i.e., anxious, depressive, and over-controlled) and externalizing behavior problems (i.e., aggressive, hyperactive, noncompliant, and under-controlled) over the past 6 months. The behavior problems are measured with 120 items on a 3-point Likert scale, consisting of ‘not true’(0), ‘sometimes true’(1) and ‘very/often true’(2). Cronbach alpha’s for the broadband scales in a Dutch sample ranged from 0.78 to 0.93 for the CBCL [57] and from 0.86 to 0.96 for the TRF [58].

### Secondary outcome measures

#### Child symptoms

*Children’s Depression Inventory* [59]. The CDI is a 27-item self-rated questionnaire that measures symptoms of depression in children (7–18 years): mood disturbances; capacity for enjoyment; depressed self-evaluation; disturbances in behavior toward other people; and vegetative symptoms, which include fatigue, oversleeping, having difficulty with activities requiring effort, and other symptoms of passivity or inactivity. Per item the child is asked to choose one of three sentences that best fits his/her feelings and thoughts in the past two weeks. The answers are calculated in a total score (ranging from 0 to 54). The internal consistency in a Dutch sample was high (α = 0.79), just as the test-retest reliability (*r* = 0.79) [60]. The CDI has high criterion validity and scores on the CDI correlate high with scores on other measures for depression [60].

*Screen for Child Anxiety Related Emotional Disorders* [61]. This questionnaire is a self-report measure that assesses anxiety symptoms in children and adolescents from age 7 on a 3-point Likert scale, ranging from ‘never, almost never’ (1) to ‘often’ (3). The scale consists of 69 items measuring symptoms of separation anxiety disorder, panic disorder, specific phobia, social phobia, obsessive-compulsive disorder, generalized anxiety disorder, post-traumatic stress disorder and acute stress disorder. The internal consistency in a Dutch clinical sample was high (α = 0.92), just as the test-retest reliability (r = 0.81) [61]. The Screen for Child Anxiety Related Emotional Disorders showed convergent validity with other anxiety questionnaires [61].

*Child Dissociation Checklist* [62] is a 20-item parent-rated questionnaire with a 3-point Likert scale answering format ranging from ‘not true’(0), ‘somewhat or somehow true’(1) and ‘very true’(2). The child dissociation checklist is a screening device and gives an indication for dissociative problems in children (5–18 years). It shows good test-retest reliability (*r* = 0.69) and internal consistency (Cronbach’s α = 0.86) [63]. Good convergent and discriminant validity have been indicated [63].

### Child adjustment

*Coping: The cognitive emotion regulation questionnaire* [64]. The cognitive emotion regulation questionnaire measures coping of younger children after stressful/negative events. In this study, we only use the subscales Rumination and Catastrophizing for children and parents. Each subscale has 4 items, and the subscales showed α = 0.79 for Rumination and 0.67 for Catastrophizing in a sample of 9–11 year old children. Children and parents rate how often they use a certain coping style on a 5-point scale ranging from ‘(almost) never’ (1) to ‘(almost) always’ (5).

*Emotional Awareness Questionnaire:* The [65] aims to identify how children and adolescents feel or think about their emotions. The questionnaire measures six aspects of emotional awareness: 1) differentiating emotions; 2) verbal sharing of emotions; 3) bodily awareness; 4) acting out emotions; 5) analyses of emotions; and 6) others’ emotions. The Emotional Awareness Questionnaire consists of 30 items on which children are asked to rate the degree to which each item is true on a 3-point scale ‘not true’(1), ‘sometimes true’(2), and ‘often true’(3). The reliability of the Emotional Awareness Questionnaire subscales ranged from 0.64 to 0.77 [65]. In a revised version ‘Acting out Emotions’ was changed to ‘Not Hiding emotions’ [66]. For the current study, we adapted the items of the Other’s Emotion subscale (5 items) such that these items enquire about parents’ emotion, not about friends’ emotions. We added items for mother and father separately (10 items). We omitted the subscale ‘Analyses of emotions’ (5 items), because this dimension was not directly associated with the aims of the treatment. This led to the inclusion of 30 items.

*Self-control: Self-Control Scale* [67, 68]. The 11-item self-control scale aims to assess parents’ and children’s ability to control their impulses, alter their emotions and thoughts, and to interrupt undesired behavioral tendencies and refrain from acting on them. For adults, the original scale shows adequate internal consistency (alphas between 0.83 and 0.85), test-retest reliability over a period of three weeks (alpha = 0.87), and validity [67]. Paralleling the findings for the English versions of the scale, the short Dutch version of the scale showed adequate reliability in earlier studies with adolescents [69]. Response categories ranged from ‘not at all’ (1) to ‘very much’ (5). In this study, we will also administer the Self-Control Scale to young children (age 7 and above) [70]. To assess perceived self-control, we will use an adapted version of the scale where each items was adjusted so as to refer to the child. Previous research shows that the scale shows good reliability [71].

*Behavior Rating Inventory of Executive Function* [72]. The BRIEF measures specific behaviors relating to executive functioning on a 3-point Likert scale, consisting of ‘never’(1), ‘sometimes’(2) and ‘often’(3). The BRIEF has a parent-report and a teacher questionnaire for 5–18 years, and a self-report questionnaire for 11–18 years. The BRIEF comprises eight clinical scales (inhibit, shift, emotional control, initiate, working memory, plan/organize, organization of materials and monitor), two composite scores (behavior regulation and metacognition) and a general executive function summary score (Global Executive Composite). The internal consistency of the Dutch BRIEF is very high (Cronbach’s alpha of the eight clinical scales ranged from 0.78 to 0.90) and the mean test-retest stability on the clinical scales was 0.81 [73].

*Fundamental needs:* To assess the four fundamental needs proposed by Williams [74], we use a measure, derived and translated into Dutch from Williams’ [74] measures. The scale measures individuals need fulfillment in general, including their *sense of belonging*, their *self-esteem*, their *sense of a meaningful existence*, and their sense of *control and agency*. For each scale, the items will be averaged and aggregated into a single score. The scale has extensively been used in research examining ostracism and has shown good psychometric properties [75]. A pilot study confirmed the usefulness and content validity of the scale with children.

*Self-esteem: Global self-worth subscale* [76]. Self-esteem will be measured using the 6-item global self-worth subscale of the Self-Perception Profile for Children [76]. This reliable scale (α = 0.72) measures the degree to which children are satisfied with themselves. Following Thomaes, Bushman, Stegge, and Olthof [77], a 4-point scale response format will be used, which ranges from ‘I am not like these kids at all’ (0) to ‘I am exactly like these kids’ (3). Higher scores indicate higher self-esteem.

### Children’s responses to IPV

*Emotional responses to IPV*: To assess children’s emotional responses to IPV, *the Security in the Interparental Subsystem* [78] will be used. The Security in the Interparental Subsystem emotional reactivity subscale has 12 items and is subdivided in four questions about emotional arousal, α = 0.74, and five questions about emotional dysregulation, α = 0.84, and three questions about behavioral dysregulation, α = 0.65. In addition to the four questions about emotional arousal (sad, scared, angry, unsafe), we will add 5 items from the Positive and Negative Affect Scale for Children [79]. These are ‘ashamed’, ‘guilty’, ‘upset’, ‘alert’, and ‘nervous’. The Security in the Interparental Subsystem uses a four-item answer format ‘not at all true of me’ (1) to ‘very true of me’ (4). Children will be asked to answer the same questions with respect to past fights and arguments between their parents and current fights and arguments between their mother and partner at T1, T2, and T3.

*Cognitive responses to IPV; self-blame, perceived threat and coping efficacy:* To assess two specific cognitions related to IPV, self-blame, and perceived threat, as well as coping efficacy with IPV, we will use the *Children’s Perception of Interparental Conflict Scale* (CPIC), developed by Grych, Seid, and Fincham [80]. Three subscales will be used: Coping efficacy, Self-blame, and Perceived Threat. The Perceived Threat subscale (6 items) assesses cognitions of perceived threat and fear. The Self-blame subscale (5 items) assesses children’s perceptions that they were responsible for causing the conflict. The Coping Efficacy subscale consists of 6 items and the respondent is asked to answer on a 3-point scale consisting of ‘true’ (1), ‘sort of true’ (2) and ‘false’ (3). At T1, T2, and T3, children will be asked only how they respond with respect to current fights and arguments. One additional question will be asked at T1, T2 and T3 about past IPV: ‘It is my fault that there were arguments and fighting between my mother and *< father/partner >’*. This questionnaire has a total of 18 items.

*Child Cognitive responses; trauma-related cognition:* To assess negative cognitive responses that are trauma-related, we will *use the Post Traumatic Cognitions Inventory – child version* which was developed by Meiser-Stedman et al. [81]. The 25 items were based on the original adult version of the PTCI [82]. The CPTCI is a reliable (α = 0.86–0.91 across three samples) measure that was originally developed for children with single-event trauma [81]. The scale assesses appraisals concerning the more abstract consequences of traumatic experiences, as well as physical threat and vulnerability. There are two subscales, ‘Permanent and disturbing change’ and ‘Fragile person in a scary world’. For this study, the word ‘event’ will be changed to ‘event(s)’ in all items to acknowledge the multiple traumatic experiences of the children exposed to IPV. The answer format consists of a 4-item Likert scale: ‘Don’t agree at all (1), ‘Don’t agree a bit’ (2), ‘Agree a bit (3), or ‘Agree a lot’ (4).

*Children’s general beliefs about aggression and family violence: Normative beliefs about aggression* (NOBAG). The 20-item NOBAG scale [83] was developed to asses children’s beliefs about the acceptability of specific aggressive behaviors in specific social contexts. The scale is divided in two parts. The 12-item subscale ‘Retaliation beliefs’ consists of short scenarios in which one child (A) is aggressive towards another child (B). The respondent is asked if it is wrong or okay for B to react with verbal aggression toward A and, second, if it is wrong or okay for B to respond with physical aggression. The second subscale ‘General beliefs’ consist of 8 items that assess general beliefs about aggression. Children can choose between four answer options: ‘Don’t agree at all’(1), ‘Don’t agree a bit’(2), ‘Agree a bit (3), or ‘Agree a lot’ (4).

*Attitudes about family violence* (AAFV) scale assesses children’s attitudes and beliefs about the acceptability of family violence [84]. The child is asked to indicate the extent to which ten statements are true on a 5-point Likert scale ranging from ‘Do not agree’(1) to ‘Strongly agree’(5). The total score indicates more negative attitudes and beliefs. The internal consistency is good [85]. For this study, the scale was translated into Dutch. The AAFV has been used in treatment studies on domestic violence [19, 86].

*Behavioral response to IPV, avoidance and approach:* The behavioral subscales of the *Security in the Interparental Subsystem* [78] will be used to assess children’s approach and avoidant behavioral responses to IPV. The subscale Involvement (α = 0.74) has 7 items and the subscale Avoidance has 7 items (α = 0.79). Involvement refers to approach behavior that the child uses to get involved in an argument between his/her parents. Avoidance refers to behavior that the child uses to escape from an argument between his/her parents. The scale uses a four-item answer format ‘Not at all true of me’ (1), ‘A little true of me’ (2), ‘Somewhat true of me’ (3) and ‘Very true of me’ (4). Children will be asked to answer the same questions with respect to past fights and arguments between their parents and *current* fights and arguments between their mother and partner at T1. At T2 and T3, children will be asked only how they respond with respect to current fights and arguments.

### Measures of mediating variables

To test whether the two specific parental components of HORIZON mediate the effects of treatment on changes in child symptoms and adjustment, measures including parental availability and parent-child interaction are administered. Those measures will also be used to test whether the two specific parental components leads to increased parental availability and to improved parenting behavior.

### Parental availability

*Security in the Family System:* The Security in the Family System scale [87] will be used to assess how much children perceive their families as a reliable source of protection, stability, and support. The subscale ‘Secure’ will be used, which assesses a secure pattern of emotional security. Children indicate the extent to which they agree with 7 statements using a four-point scale ranging from ‘Complete disagree’(1) to ‘Complete agree’(4). Psychometric properties of this security subscale are good, Cronbach’s α = 0.85 and test-retest reliability = 0.82 [87].

*The cognitive emotion regulation questionnaire* [88] is a self-report measure developed in the Netherlands that assesses cognitive coping-styles of adults and adolescents aged 12 years and older. Two subscales of the cognitive emotion regulation questionnaire are included in this study: rumination and catastrophizing*.* Each subscale has 4 items, and has good internal consistency (α = 0.83 for rumination and α = 0.79 for catastrophizing [88]. The parent rates how often he or she uses a certain coping style on a 5-point scale ranging from ‘(almost) never’(1) to ‘(almost) always’(5). We use two versions of the questionnaire, one with the original questions and one version adapted for this study. The adapted version asks parents to report on how they cope with the traumatic events that happened to their children.

*Emotional Awareness: The Emotional Awareness Questionnaire* [65] is described in more detail above. For parents, we will include two subscales: “Not Hiding Emotions” and “Other’s Emotions”. We changed the items belonging to the Other’s Emotions subscale to enquire about children, not friends.

*Daily Psychological Availability Scale* [89]. To assess parental availability for the child we will use eight adapted items of the Daily Psychological Availability Scale. Items were measured using 7-point scales from 1 (totally disagree) to 7 (totally agree). Cronbach’s α was 0.78 for both fathers and mothers [89]. A higher score on this scale represents more psychological availability for the child.

*The Children’s Responses to Trauma Inventory Revised version* [90] is a self-report measure for children aged 8–18 years and consists of 34 items. Children answer, on a 5-point Likert scale, to which extent a reaction to a traumatic event was present during the past week. The instrument has 4 subscales: intrusion, avoidance, arousal, and other child-specific responses (e.g., feelings of guilt, regressive behavior, reckless behavior, fear of the dark, fear of going to the toilet at night, separation anxiety, sadness, crying, feeling tired, and psychosomatic complaints). A recent study found good psychometric properties for the scale, with Cronbach’s α of 0.92 for the total scale, and α ranging from 0.72 to 0.81 for the four subscales [91]. The parent-reported questionnaire is intended for parents of children age 4 to 18 years. The scale consists of the same 34 items measuring intrusion, avoidance, arousal, and nonspecific symptoms during the last week. Previous research shows sufficient reliability (α total scale = 0.92) and good convergent and discriminant validity [92]. To assess concordance between parent- and child-reported trauma symptoms and parental insight in the child’s trauma symptoms, we will instruct parents to fill out the questionnaire how they think the child will answer and will compare child and parent forms. This information is obtained at session 1, 9 and 15.

*During the therapy*, in session 6, children will be asked to draw and talk about the most adverse incident they can remember about the IPV. This is part of the regular treatment protocol of the HORIZON for children. In addition, in session 6, we will ask the parents in the parallel group to write down what they think their child will talk about with respect to the most aversive IPV incident. The answers to this question from both parent and child will be compared. One of the aims of the preparatory program is to coach parents to differentiate between their own trauma-history and the one of the child. If this aim is achieved, we hypothesize that parents in treatment condition 1 & 2 (see Fig. 1) will more often provide an answer that is similar to their child’s to the above question than the parents in treatment condition 3 & 4 (see Fig. 1).

### Parent-child variables

*Attachment Security: The Security Scale* [93] is a 15-item self-report questionnaire for children between the ages of 8 and 18 and measures attachment security with their parents. Children report on their attachment security with both parents separately. The scale consists of three dimensions: perceptions of responsiveness and availability of the parent, tendency to seek parental support in times of stress and the quality of communication with the parent. Children rate the items on a 5-point Likert-Scale ranging from ‘strongly agree’ (1) to ‘strongly disagree’ (5) with a higher score indicating greater perceived attachment security. Several studies indicate adequate reliability and validity [94]. Kerns, Tomich, Aspelmeir and Contreras [95] reported high internal consistencies for 10 and 12 year olds (α = 0.82, α = 0.79). Test-retest reliability over a two-week period was high (r = 0.75) [93]. The Security Scale is related to other attachment measures [95]. In a Dutch sample, internal consistency was α = 0.77 for the mother version and α = 0.85 for the father version [96].

*Generalized Trust Beliefs: Children’s Generalized Trust Beliefs* [97]. The original questionnaire assesses children’s generalized trust beliefs across three bases of trust (reliability, emotionality and honesty) in four target groups (mother, father, teacher and peers). Reliability measures the belief that parents will keep their promises. Emotionality measures the belief that parents keep their secrets confidential. Honesty measures the belief that parents are truthful. For the purpose of this study, we only use the mother and father as targets, which results in a total of 12 items. Names and situations were adjusted to fit the Dutch population. Children are presented with specific situations of fictional children and instructed to imagine that they were the children in the stories. Items are rated on a 5-point Likert scale ranging from ‘very unlikely’ (1) to ‘very likely’ (5). Reliability and validity were acceptable in earlier research (total scale 0.76, reliability subscale: 0.67, emotionality subscale: 0.62, honesty subscale: 0.65) [97]. A pilot study confirmed the usefulness and content validity of the scale with children.

To assess parenting behavior, both from the child’s and the parent’s perspective, we will use *the Ghent Parental Behavior Questionnaire* [98]. The brief child version of the GBPS has 25 items for each parent [99]. The parent version of the GBPS has 60 items and includes nine subscales: Positive parenting, monitoring, rules, discipline, inconsistent discipline, harsh punishment, ignoring, material rewarding, and autonomy. Cronbach’s α for the subscales is moderate to good [98].

*Capitalization Scale* [100]. To assess parents’ capitalization attempts to their child’s sharing of positive events, we adopted Gable et al.’s [100] capitalization scale. Parents indicate, using 5-point scales, whether they “reacted enthusiastically to their child’s sharing of a good event” (Active–Constructive), “pointed out the potential problems or down sides of the good event” (Active–Destructive), “said little, but my child knew I was happy for him/her” (Passive–Constructive), and “seemed disinterested” (Passive–Destructive). The original scale was found to be reliable and valid [100].

*Self-Control:* For a description see child adjustment measures above.

*Protective Factors Survey* [101]. The Protective Factors Survey assesses multiple protective factors against child maltreatment. The survey consists of 20 items and five subscales, namely Family Functioning/Resiliency, Child Development/Knowledge of Parenting, Concrete Support, Social Support and Nurturing and Attachment. Items are rated on a (7-point) frequency or agreement scale.

*The Confusion Hubbub and Order Scale* [102] measures environmental confusion in the home. This refers to the levels of environmental noise, crowding and disorganization in a household, in short: chaos. The questionnaire was translated into Dutch for this study, and includes 15 statements that are rated by the parents as ‘not true’, ‘quite true’ or ‘very true’. The Confusion Hubbub and Order Scale has good internal consistency (α = 0.79) and test-retest reliability (α = 0.74) [102].

*The Inclusion of the Other in the Self Scale* [103]. This Inclusion of the Other in the Self Scale is a single-item pictorial measure for closeness to others. Parents and children describe their relationship by selecting a set of two circles. The degree of overlap between the circles stands for the degree of closeness to the other person. Parents rate on the relationship with their child and children rate on their relationship with the mother and father separately. Respondents are presented with 7 different sets of circles, ranging from a set with no overlap and a long distance between the circles and a set with almost complete overlap between the circles. The circles are held constant and only the overlap increases. Validity and reliability were adequate [103]. The Inclusion of the Other in the Self Scale has not been used before to measure the parent-child relationship. Our pilot study confirmed the usefulness and content validity of the scale with children.

*Time spent together:* Parents and children are asked 9 questions about the time they spent together during the past week. For example: “How often did you have breakfast together with your mother/child last week”. Items are rated on an 8-point scale ranging from ‘not at all’, ‘one time’, to ‘every day’.

### Treatment fidelity

All treatment sessions are audio or video-recorded to ensure that the treatment protocol of the HORIZON [10, 44] was followed. Tapes are randomly selected to be coded for treatment adherence.

### Control measures

*Parental psychopathology symptoms.* To assess parental psychopathology, we will use the *Impact of Events Scale – Revised* [104]. The Dutch version ‘*Schokverwerkingslijst* (SVL-22)’ was developed by Kleber and De Jong [105]. This questionnaire consists of 22 items measuring symptoms of post-traumatic stress disorder during the last week. The Schokverwerkingslijst-22 measures three dimensions: intrusion, avoidance and hyper-arousal. Parents rate the items on a 5-point Likert-scale ranging from ‘not at all’ to ‘extremely’. The measure has high internal consistency (α = 0.88) [106]. Further, we will use the *Young Adult Self-Report* [107]. The Young Adult Self-Report will be used to assess psychopathology symptoms in parents. This questionnaire has the same format as the CBCL described above. The short version of 29 items will be used in this study to limit the amount of time needed to fill in the questionnaire. Previous research has shown that these items discriminated well between referred and non-referred subjects [108]. Items are rated on a 3-point scale ranging from ‘not true’ (0), ‘somewhat or sometimes true’(1) and ‘very true or often true’(2). Reliability and validity of the Dutch version are good [109].

*Insightfulness Assessment* [110] (only at T1). The Insightfulness Assessment is based on a semi-structured interview that evaluates the parents’ ability to seek explanations regarding the motives underlying their children’s behaviors and to talk about them in an open, complex, insightful, and accepting manner. The interview is based on the parent viewing child-parent or child-examiner interaction segments which are video-taped beforehand and answering questions about the segments. The interview transcripts will be coded on 10 rating scales and classified into one of the following four categories: Positively insightful, One-sided, Disengaged and Mixed. This represents a constellation of parental thoughts, feelings, and perceptions regarding the child’s inner experience. Each transcript is independently coded by two coders blind to any information about the study. In a previous study, inter-rater reliability ranged from 0.77 to 0.93; inter-rater reliability on the four-way Insightfulness Assessment classification system was 0.84 [36].

*Autobiographical Emotional Events Dialogues* [111] (only at T1). In the AEED parents and children participate in an emotion discussion task, in which they recall and describe an event when the child felt happy, sad, mad, and scared, respectively. They are asked to jointly describe the event and to talk about what the child felt, thought, and did during the event. Construction of the stories is scored on 7 parent scales (Shift of focus, Boundary dissolution, Acceptance and tolerance, Hostility, Involvement and reciprocity, Closure of negative feelings and Structuring of the interaction) and 7 child scales (Shift of focus, Boundary dissolution, Acceptance and tolerance, Cooperation and reciprocity, Resolution of negative feelings and Elaboration of the stories). Also, the stories are scored on adequacy of the stories and coherence. Rating scales range from 1 to 9, a higher score indicates a greater presence of that particular construct. Ratings are based on the complete session. Scores result in a classification in one of four categories: emotionally matched, emotionally unmatched: excessive, emotionally unmatched: flat or emotionally unmatched: inconsistent. Each videotaped session is independently coded by two coders (research assistants). Coders are blind for experimental condition. In a previous study, inter-rater reliability ranged from 0.87 to 0.95 [112].

*Positive filler task*: To ensure all dyads end the AEED with a positive task before they continue with the Family interaction task, we will ask the child to briefly talk about his/her favorite food or hobby with his/her parent.

*Family interaction task* [113] (only at T1). This observational instrument measures parent-child interaction and consists of four tasks in which parent and child are instructed to complete a series of interactive tasks together. The first task is a word guessing game, in which parent and child take turns in guessing what word/picture appears on the card of the other. The second task involves getting marbles into designated holes in a labyrinth. In the third task, parent and child have to plan a pretend birthday party. The final task is constructing different patterns of pieces that match given designs. Nine rating scales are used for this study: 3 parent scales (positive responsiveness, anger and hostility, quality of assistance), 3 child scales (persistence and diligence; anger, defiance and frustration; expression of positive affect) and 3 dyadic scales (collaboration and teamwork; positive affect; negative affect/conflict). Rating scales range from 1 to 5 and a higher score indicates a greater presence of that particular construct. Ratings are based on the complete session. Each videotaped session is independently coded by two coders (research assistants). In a previous study, inter-rater reliability ranged from 0.63 to 0.73 [113].

*Severity and intensity of IPV.* To assess the severity and intensity of the violence that children have been exposed to, we will use a combined measure including items from the Conflict Tactics Scale [114], the Conflict Tactics Scale parent child [114], the Parents Report of Traumatic Impact [115]; and the Adverse Childhood Experience Questionnaire [116]. This combined questionnaire has been used in a previous study on the effectiveness of a psycho-educational prevention program for children exposed to IPV [117]. The questionnaire covers the topics of duration of the violence, the nature of the arguments in the relationship with the (ex-partner), followed by items from the Conflict Tactics Scale parent child and Parents Report of Traumatic Impact about problems between parent and child, and traumatic events the child has experienced. The questionnaire also includes items about traumatic experiences in parents’ own childhood.

*New IPV incidents:* Parents and children are asked 8 questions if any new IPV incidents or other stressful events occurred.

### Statistical analyses and sample size calculation

All variables are measured on at least an ordinal scale. All scale scores will be examined for normality. Should we observe deviations, steps will be taken to ensure optimal estimation of parameters in our analyses. The proposed study includes a nested structure, because individuals are treated within groups. Following the recommendations by Peugh [118], we will calculate design effects to assess group-level dependency and examine whether multi-level modeling is required. To ensure comparability of the randomized conditions, we will control for duration and severity of the IPV [45], parental psychopathology [46], and new incidents of IPV. Power calculations were performed using the program G*power 3 [119], assuming that individual-level effects can be treated as independent data. We determine required sample size to be 100 parent-child dyads, achieving statistical power of at least .80 for the primary and secondary objectives, as described in more detail in the following paragraphs. We will test for the randomness of missing data. If this is the case we will use multiple imputation.

### Primary objective

The first goal of the present study is to examine the efficacy of two parental components that complement a TF-CBT-based core treatment for children who have been exposed to IPV. Therefore, a multiple regression analysis will be executed with two dummy variables as independent variables, representing both parental components (dummy 1 = parent preparatory program present vs. absent; dummy 2 = parent-child interaction sessions present vs. absent) and their interaction. Dependent variables are child symptoms (primary outcomes) at the three time points (T1, T2, and T3) clustered into 1) Trauma symptoms, 2) Internalizing symptoms, 3) Externalizing symptoms. Analyses will be independently executed for the three dependent variables. Because children in each group receive intervention, a medium effect size is expected (f^2^ = .15). With a maximum of five predictors and an alpha of .05, we achieve a power of .84 with 100 children included in the study.

### Secondary objectives

To investigate associated changes between: child symptoms, on the one hand, and 1) child responses (i.e., child emotional, behavioral, and cognitive responses), 2) parental availability and 3) parent-child interaction on the other, we will use the stepwise procedure in multivariate regression analyses with a maximum of 4 tested predictors. With a sample size of 100, alpha of .05 and medium effect size (f^2^ = .15), we will achieve a power of .87 in analyses with 4 individual predictors. We will use latent variables for child responses, parental availability and parent-child interaction.

We will also use multivariate regression analyses to study the fourth goal, namely which mechanisms explain how the two components added to the TF-CBT-based Horizon treatment contribute to the effectiveness of TF-CBT-based Horizon treatment. We will follow Holmbeck’s recommendations [120] for these mediational analyses and will test the robustness of our results by using the bootstrapping method. These analyses will allow us to determine whether the parent-child interaction variables and parental availability partly or fully mediate the effects of each parental component on child symptoms. Specifically, we will test whether, as predicted, the preparatory program leads to increased parental availability and whether the parent-child interaction sessions lead to improved parenting behavior. Also, we will explore whether these changes lead to a reduction in symptoms. Because at least partial mediation is assumed, a small to medium effect size is chosen (f^2^ = .10). Two models are tested. A first model with component 1 (preparatory parent program) and parent availability as independent variables. And a second model with component 2 (parent-child interaction sessions) and parent-child interaction as independent variables. With a sample size of 100, alpha of .05 and medium effect size (f^2^ = .10), we will achieve a power of .80 if 2 predictors are tested in the regression.

*Exploratory analyses: Cross-lagged panel model.* Based on the recommendations of Kazdin [47] to investigate mediators and mechanisms of change in intervention RCT’s, to test the causal direction of the longitudinal relation between the different types of mediators and child outcomes, we will conduct cross-lagged panel analyses [121]. The model will include three waves of child symptoms and parent-child variables, parental availability, and child responses, respectively. We will estimate T1 associations (interpreted as correlations at T1), T2 and T3 stability (interpreted as relative stability over time), correlated change (interpreted as overlapping relative change in two variables), and cross-lagged paths between child symptoms and parent-child variables, parental availability, and child responses, respectively (interpreted as a linkage of the level of one variable at a given time point with a relative change in another variable one assessment later). Correlated change and cross-lagged paths reflect longitudinal relationships, and will be interpreted as such. These cross-lagged analyses will also be carried out with the repeated measures at session 1, 9 and 15 for child trauma symptoms, closeness and time spent together with possibilities to explore more than three waves of measurements.

### Handling and storage of data and documents

Privacy of participants will be protected by allocating identification numbers to the personal information, which will be traceable with a separate list. This list with personal information (names, addresses, phone numbers) that connects the participants with the research data, is accessible to one of the researchers and will eventually be destroyed. Data will be analyzed in a way that no conclusions can be drawn about individual participants.

The research data will be stored and managed by the research team. All employees who work with confidential data will sign a confidentiality agreement, on which they state not to share the information with third parties. Only if the safety of a parent or child is in danger, these concerns will be shared with the participating organization where the program is carried out.

The research material and the confidentiality agreements will be stored, according to the publication manual of the American Psychological Association, in a locked file cabinet at the VU University for five years after the last publication based on this data.

### Public disclosure and publication policy

The research data will be published in international and national journals, and all affiliated organizations will be mentioned. The results will also be presented on international conferences. The clinical trial is registered at the Dutch trial register (http://www.trialregister.nl/trialreg/admin/rctview.asp?TC=4015). To make the results also available for Dutch policy makers and service providers, we plan to publish the results in (national and international) journals within the field of youth mental health care.

### Ethical considerations

The study protocol has been approved by the Medical Ethics Committee of the VU University Medical Center in Amsterdam, the Netherlands (METC VUmc 2011/101/NL39277.029.12). All substantial amendments will be presented to the METC and to the competent authority. Non-substantial amendments will not be notified to the accredited METC and the competent authority, but will be recorded and filed by the sponsor, ZonMw, the Dutch organization for healthcare research and innovative care. All changes will be described and discussed in the publications of the study results.

The HORIZON has been used to treat children who have experienced IPV for more than 10 years in several children and youth treatment centers in the Netherlands and does not seem to involve risks for participants. Nevertheless, should a child or a parent seem adversely affected by therapy, questionnaires or observational tasks as observed by the researchers or therapists, it may be decided to (temporarily) discontinue participation in this study. Participants can leave the study at any time for any reason if they wish to do so without any consequences. Withdrawal of participants from the study will have no impact on their treatment. Participants who withdraw from the study will not be replaced, because there is no place for them in the therapy groups, and it is not possible for parents and children to start at a later time during treatment. Should participants withdraw from the treatment, these parents and children will be followed according to the intention to treat principle. They will be asked fill in the questionnaires and the observational tasks.

## Discussion

This study aims to bridge the gap between clinical practice and scientific research [122]. It examines mechanisms underlying change in psychotherapy for children exposed to IPV, explains how components of therapy work, and identifies moderators of treatment effects. The results, which are obtained in a RCT-study in collaboration with different trauma centers, will provide unique insights to improve clinical decision-making.

First, the RCT-design provides the best possible controls for evaluating the efficacy of psychiatric treatment. Rather than using a waiting list control group, the study zooms in on two parental components and mechanisms of change not yet investigated. All children receive TF-CBT, an established treatment for traumatized children [17], including psycho-education, parenting skills, relaxation, affective expression and modulation, trauma narrative, and cognitive coping and processing.

Second, the study focuses on the efficacy of two IPV-related parental components added to TF-CBT. To date, research on effective components of treatment for post-traumatic stress disorder focused on trauma-oriented components [123]. Research on effective components of TF-CBT focused on the trauma-narrative [124], on treatment lengths [124], and on inclusion of parents [125]. Nevertheless TF-CBT is less effective for children exposed to IPV than for children otherwise traumatized [9]. Children exposed to IPV are not only traumatized, but IPV also directly and indirectly affects their emotional, cognitive and behavioral responses. By considering this complexity this study provides important insights into the efficacy of specific IPV-related parental components. These insights are crucial to improve the treatment and maximize its effects for this specific target group [124].

The use of multiple informants (parent, child and teacher) and independent observations and interviews are likely to diminish reporting-bias. To pinpoint underlying mechanisms and assess the longer-term consequences of the intervention multiple data collections takes place, including a follow-up at 6 months after the end of treatment. This converging evidence will allow us to establish the reliability and validity of the collected information considerably.

A specific limitation of research on child abuse in The Netherlands is that by Dutch law, both custodial parents have to give informed and written consent to participation of their child in the study. Conflict between parents may extend to conflict about the participation of the child in treatment and scientific research, which may bias the sample of participants.

In short, the current RCT-study will enhance our understanding of the efficacy IPV-related parental components added to TF-CBT for children who have been exposed to IPV. It will illuminate mechanisms underlying change by considering multiple dimensions of child responses.

## Trial status

The study protocol has been approved by the Medical Ethics Committee of the VU University Medical Center in Amsterdam, the Netherlands (METC VUmc 2011/101/NL39277.029.12). We just started to include children and parents and will be doing so for the coming four years. We expect the main results to be published in 2017.

## Abbreviations

AEED: Autobiographical Emotional Events Dialogue
BRIEF: Behavior Rating Inventory of Executive Function
CBCL: Child Behavior Checklist
CDI: Children’s Depression Inventory
IPV: Interparental violence
METC: Medical Ethics Committee
RCT: Randomized clinical trial
TF-CBT: Trauma Focused-Cognitive Behavioral Therapy
YSR: Youth Self Report
